# A Flavonoid Glycoside Compound from *Siraitia grosvenorii* with Anti-Inflammatory and Hepatoprotective Effects In Vitro

**DOI:** 10.3390/biom14040450

**Published:** 2024-04-07

**Authors:** Juanjiang Wu, Huaxue Huang, Limin Gong, Xing Tian, Zhi Peng, Yizhun Zhu, Wei Wang

**Affiliations:** 1 Faculty of Chinese Medicine, Macau University of Science and Technology, Macau SAR 999078, China; 3230004587@student.must.edu.mo (J.W.); 2 TCM and Ethnomedicine Innovation & Development International Laboratory, Innovative Materia Medica Research Institute, School of Pharmacy, Hunan University of Chinese Medicine, Changsha 410208, China; 3 Hunan Huacheng Biotech, Inc., High-Tech Zone, Changsha 410205, China; pengzhi3778@163.com

**Keywords:** *Siraitia grosvenorii*, flavonoid glycoside, anti-inflammatory, hepatoprotective

## Abstract

Inflammation is a pivotal factor in the development and advancement of conditions like NAFLD and asthma. Diet can affect several phases of inflammation and significantly influence multiple inflammatory disorders. *Siraitia grosvenorii*, a traditional Chinese edible and medicinal plant, is considered beneficial to health. Flavonoids can suppress inflammatory cytokines, which play a crucial role in regulating inflammation. In the present experiments, kaempferol 3-*O*-*α*-L-rhamnoside-7-*O*-*β*-D-xylosyl(1→2)-*O*-*α*-L-rhamnoside (SGPF) is a flavonoid glycoside that was first isolated from *S*. *grosvenorii*. A series of experimental investigations were carried out to investigate whether the flavonoid component has anti-inflammatory and hepatoprotective effects in this plant. The researchers showed that SGPF has a stronger modulation of protein expression in LPS-induced macrophages (MH-S) and OA-induced HepG2 cells. The drug was dose-dependent on cells, and in the TLR4/NF-κB/MyD88 pathway and Nrf2/HO-1 pathway, SGPF regulated all protein expression. SGPF has a clear anti-inflammatory and hepatoprotective function in inflammatory conditions.

## 1. Introduction

Inflammation treatment has received considerable scholarly attention in recent years, and researchers have shown increased interest in the topic. Inflammation is a complex and ever-changing process in which some molecules, called pro-inflammatory cytokines, including TNF-α, IL-6, IL-1β, and VEGF, have crucial impact [1]. LPSs are potent immune cell activators. Immune cells require immunogenic stimulants such as LPSs to generate inflammatory cytokines. The cell surface receptor complex TLR-4, when activated by LPSs, initiates a specific intracellular route. This process involves the binding and activation of the adaptor molecule MyD88 [2]. Hence, researchers have proposed the ’three-hit’ process as a provisional description of the pathological development of NAFLD, involving steatosis, oxidative stress, and inflammatory mediators such as TNF-α and IL-6 [3]. There is an increasing recognition that inflammation and oxidative stress are closely related. Patients experience increased levels of various inflammatory mediators compared to individuals without NAFLD [4]. In addition, acute inflammatory reactions, the process of aging, and/or mechanical loading can all contribute to increased levels of oxidative stress, resulting in a breakdown in antioxidant enzyme expression and ROS scavenging systems [5]. Nrf2 is an important transcription factor classified under the cap ’n’ collar subfamily [6]. Nrf2 can regulate genes that are antioxidative enzymes and play a protective role against oxidative stress through regulating antioxidative genes, such as HO-1 [7]. The inflammatory response is a pre-reaction to most illnesses and can alert the person to seek medical attention promptly, and should not be ignored. Healthcare professionals commonly employ steroidal and non-steroidal anti-inflammatory medicines in therapeutic settings. However, it is important to note that the safety profiles of these treatments vary among individuals and may result in significant incidence of adverse effects. Therefore, finding more effective and safer anti-inflammatory compounds for clinical use is one of the hot spots in medicine research, and the inclusion of traditional Chinese medicine could help to expand the research scope. There have been a growing number of reports indicating that herbal extracts have demonstrated anti-inflammatory properties both in vitro and in vivo [8].

*Siraitia grosvenorii* is a perennial herbaceous vine; the formal Chinese name of *S. grosvenorii* is Luohanguo (罗汉果) [9]. It is an herb which is utilized for both food and medicine in China. The fruit of *S. grosvenorii* is edible, with a unique flavor and pungent taste, and not only improves appetite as a sweetener, but also has an anti-inflammatory effect, and is one of the common products on the tea table; its fruit also can be used as medicine for clearing phlegm, relieving coughs and asthma, relaxing the bowels to relieve constipation, and so on [10]. Notably, *S. grosvenorii* has earned official recognition as both medicine and food, as stipulated by the National Health and Wellness Commission in China, attesting to its elevated status in terms of both edibility and medicinal efficacy. Oral administration of NHGR significantly reduced epidermal hyperplasia and inflammatory cell infiltration in skin lesions from DfE-induced atopic dermatitis and reduced serum immunoglobulin E levels [11]. The fruits of *S. grosvenorii* yielded mogrosides, glycosides responsible for sweetness, which are considered the primary active ingredients contributing to its natural sweetness and biological function. Existing studies have shown that the extracts and individual compounds of *S. grosvenorii* are non-toxic [12], and have anti-inflammatory, antioxidant, antidiabetic, anti-COVID-19, liver-protective, and other biological properties [13,14,15,16]. Asthma is a chronic airway disease that has historically been classified predominantly as an inflammatory disorder [17]. As a result, current therapeutic interventions focus on addressing inflammation. The mechanism of Chinese medicine constituents in treating asthma mainly involves the regulation of the immune system, suppression of the inflammatory response, and relaxation of the bronchial airways to achieve relief from asthma symptoms [18]. Administration of mogroside V effectively attenuated OVA-induced airway hyperresponsiveness and reduced the number of inflammatory cells in BALF. Mogroside V has shown histological evidence of reducing the inflammatory infiltrate in the lungs of asthmatic mice [19]. These findings make an important contribution to establishing the fact that many constituents in *S. grosvenorii* have anti-inflammatory activity, primarily belonging to triterpenoid glycosides known as mogrosides. Similarly, in order to find other anti-inflammatory compounds also present in *S. grosvenorii*, the researchers chose to study the constituents with the second-most abundant, flavonoids.

Scientific data are increasingly indicating that flavonoids possess anti-inflammatory and hepatoprotective effects. These substances possess strong anti-inflammatory properties and decrease inflammatory damage to tissues. However, few flavonoids were isolated from *S. grosvenorii*, and only a few had anti-inflammatory activity. Flavonoids obtained from *S. grosvenorii*, such as kaempferol, exhibit anti-inflammatory and hepatoprotective properties. They can decrease the expression of pro-inflammatory cytokines, downregulate inflammatory markers, and prevent inflammatory damage [20]. Flavonoids have been found to possess the ability to hinder regulatory enzymes or transcription factors, and are also abundant in foods. Thus, multiple in vitro and in vivo studies have demonstrated that flavonoids possess the capability to impede the initiation and advancement of inflammatory diseases [21].

**Figure 1:**
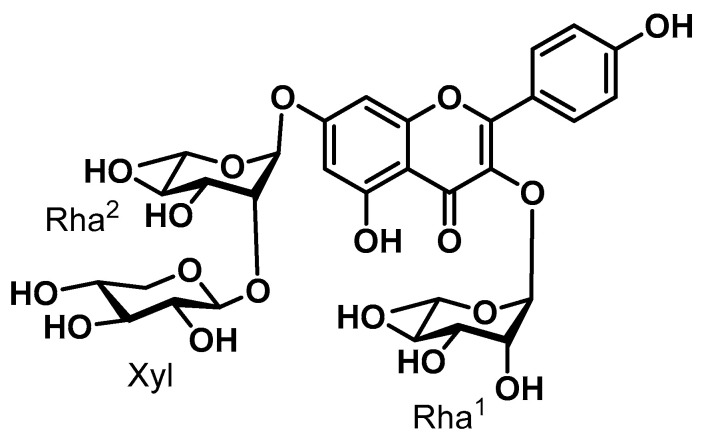
The molecular structure of kaempferol 3-*O*-*α*-L-rhamnoside-7-*O*-*β*-D-xylosyl(1→2)-*O*-*α*-L-rhamnoside (SGPF).

SGPF (*S. grosvenorii* pomace flavonoid), named kaempferol 3-*O*-*α*-L-rhamnoside-7-*O*-*β*-D-xylosyl(1→2)-*O*-*α*-L-rhamnoside, is a natural substance isolated from *S. grosvenorii* which was obtained for the first time from the genus *Siraitia* and family Cucurbitaceae; the anti-inflammatory mechanism of this compound has not been fully elucidated. To further verify its anti-inflammatory activity and enrich the types of anti-inflammatory constituents in this plant, a series of experiments were conducted on SGPF (Figure 1).

## 2. Materials and Methods

### 2.1. Materials

MH-S and HepG2 cells were obtained from Procell Life Science & Technology Co., Ltd. (Wuhan, China); kaempferol 3-*O*-*α*-L-rhamnoside-7-*O*-*β*-D-xylosyl(1→2)-*O*-*α*-L-rhamnoside (PubChem CID: 74978103, SGPF) was obtained from the TCM and Ethnomedicine Innovation & Development International Laboratory Innovative Materia Medica Research Institute, School of Pharmacy, Hunan University of Chinese Medicine (Changsha, China), the extract was identified by Prof. Wei Wang, and a specimen (no. 20200911) was deposited in the lab; RPMI1640 Medium (RP-1640), MEM (obtaining NEAA), fetal bovine serum (FBS), trypsin, penicillin/streptomycin, and PBS were obtained from Procell (Wuhan, China); lipopolysaccharides (LPSs) were obtained from Sigma (St. Louis, MO, USA); oil acid (OA) was obtained from Macklin Co. Ltd. (Shanghai, China); CCK-8 was purchased from Seven Biotech (Beijing, China); RIPA buffer and PMSF were purchased from Solarbio (Beijing, China); TNF-α, IL-1β, IL-6, and IL-10 ELISA kits were purchased from Bioswamp (Wuhan, China); and a tissue total cholesterol assay kit, tissue total triglyceride assay kit, liquid sample MDA assay kit, and tissue SOD assay kit were purchased from Applygen Technologies Co., Ltd. (Beijing, China).

### 2.2. Isolation and Purification of SGPF

A total of 900 g of the *S. grosvenorii* pomace was dispersed in 700 mL of water and extracted by PE, EA, and n-BuOH in turn, yielding a 193.86 g EA layer. The ethyl acetate layer was submitted to a silica gel column (100–200 mesh, 10 cm × 60 cm) and eluted with DCM-MeOH (10:1→7:1→5:1→3:1→1:1, *v*/*v*) gradiently, and then detected on TLC. Fr.K was then subjected to reversed-phase column chromatography (10–60% MeOH), and the obtained flow was purified by recrystallization to afford SGPF (45.0 mg).

### 2.3. Cell Culture

Mice MH-S cells were cultured in RP-1640 containing 10% FBS, 1% penicillin/streptomycin, and 0.5 μM β-Mercaptoethanol [22], and human HepG2 cells were cultured in MEM (NEAA) containing 10% FBS and 1% penicillin/streptomycin [23] in an incubator at 75% humidity, 37 °C, and 5% CO_2_. The culture medium was changed 2-3 times a week. When cells reached 80–90% confluence, 0.05% trypsin–EDTA was used for digestion and passaging at a volume ratio of 1:2.

### 2.4. Inhibited Proliferation Activity against MH-S and HepG2: Bioassay Cytotoxicity Analysis

The inhibited proliferation activity of SGPF against MH-S and HepG2 cells was evaluated by the standard CCK-8 assay methods. Two cells were seeded in 96-well plates at a density of 1 × 10^4^ cells/well and treated with SGPF (0, 2.5, 5, 10, 15, and 20 μM) for 24 h, followed by 10 µL of CCK-8 being added to each well and incubated for 30 min. The absorbance was measured at 450 nm. An 800TS microplate reader (Agilent, Santa Clara, CA, USA) tested the data.

### 2.5. Cytokine Analysis with ELISA Kits

The MH-S cell supernatants were divided into the NC group, the 100 ng/mL LPS group, and different administration concentration groups. The levels of TNF-α, IL-1β, IL-6, and IL-10 in cell cultures were measured using ELISA (enzyme-linked immunosorbent assay) kits according to the instructions of the manufacturer. Data are shown in picograms per milligram of protein (pg/mg prot).

### 2.6. Oil Red O Staining

The formation of lipid droplets in HepG2 cells was assessed using Oil Red O staining according to methods described in the literature [24]. HepG2 cells were washed with room temperature PBS and then fixed with 4% Paraformaldehyde Fix Solution for about 30 min. After fixation, they were rinsed in 60% isopropanol once and stained with Oil Red solution for 30 min. The accumulation of intracellular lipids was observed under the microscope and recorded. 

### 2.7. Total Cholesterol and Total Triglyceride Levels in Cells

After drug administration for 24 h, 0.5 mM oleic acid (OA) was incubated for 24 h, the medium was discarded, the cells were washed with PBS, and the adherent cells were digested; then, the culture solution was added and transferred to a centrifuge tube. After centrifugation, the cell precipitate was washed with PBS. To make the cell precipitate, 50 μL cell lysis buffer was mixed in the refrigerator at 4 °C for 30 min. When the cell lysis was complete, the supernatant was determined according to the instructions. The remaining cell lysate was taken as a supernatant for the BCA kit.

### 2.8. Intracellular GPx and SOD Content Test

After administration for 24 h, 0.5 mM OA was incubated for 24 h. The medium was discarded, and the cells were washed with PBS, collected, and centrifuged; then, 50 μL lysis buffer was added and placed at 4 °C for 30 min. After centrifugation, the supernatant was taken, and the kit was used to detect the GPx levels and the SOD level by applying the WST-8 method.

### 2.9. Intracellular MDA Content Test

After administration for 24 h, 0.5 mM OA was incubated for 24 h. The medium was discarded, and the cells were washed with PBS, collected, and centrifuged; then, 50 μL lysis buffer was added and placed at 4 °C for 30 min. After centrifugation, one supernatant was taken, and the MDA level was detected; the other was taken as a supernatant to the BCA kit.

### 2.10. Intracellular Reactive Oxygen Species (ROS) Level

The cell precipitates were mixed with diluted DCFH-DA and placed in a 37 °C, 5% CO_2_ incubator for 30 min. Then, the cells were washed with DMEM to sufficiently remove DCFH-DA that did not enter the cells, and the level of ROS production was measured by the kit. The data were measured using a RF-6000 fluorescence microplate reader (Shimadzu, Kyoto, Japan).

### 2.11. Western Blot Analysis

The proteins were extracted with RIPA lysis buffer and PMSF; then, the protein quantification was conducted with a BCA protein quantification kit. Processed protein samples were collected for 8–10% SDS-PAGE electrophoresis and transferred on PVDF membranes. The membranes were blocked with 1× TBST containing 5% skimmed milk powder for 1 h at room temperature. After membrane washing, the membranes were incubated with the primary antibodies listed below at a temperature of 4 °C overnight: anti-TLR4 (1:1000, Protein-tech, Wuhan, China), anti-NF-κB (1:1000, Protein-tech, Wuhan, China), anti-MyD88 (1:1000, Protein-tech, Wuhan, China), anti-AQP1 (1:1000, Protein-tech, Wuhan, China), anti-HO-1 (1:1000, CST, Boston, MA, USA), anti-Nrf2 (1:1000, Protein-tech, Wuhan, China), and anti-β-actin (1:1000, Affinity, Changzhou, China). The membranes were then incubated with horseradish peroxidase (HRP)-labeled goat anti-rabbit/mouse antibodies (1:3000, protein-tech, Wuhan, China) for 1 h at 37 °C, and the membranes were rinsed 6 times with 1× TBST to remove any remaining antibodies (5 min every time). The images were captured using an Automatic Electrophoresis Gel Imaging System (Tanon 5200 Multi, Shanghai, China).

### 2.12. Statistical Analysis

All experimental data were expressed as the mean ± standard deviation of the mean. The treatment effects were compared by one-way analysis of variance (ANOVA) followed by Tukey’s test. *p* < 0.05 was considered statistically significant. The drawing was performed using GraphPad Prism 8.4.0 software.

## 3. Results

### 3.1. Results of CCK-8 Assay

**Figure 2:**
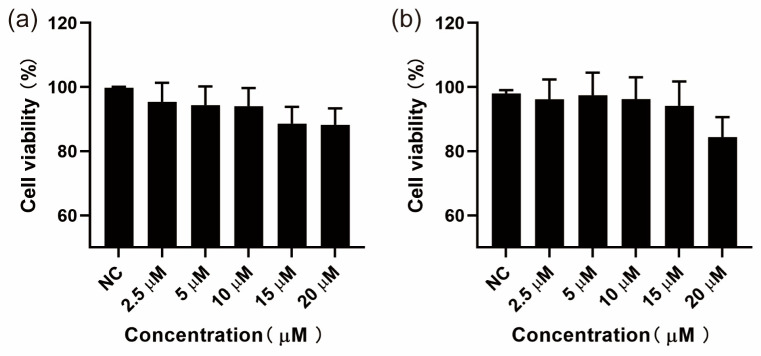
Effect of SGPF on the cell viability of MH-S and HepG2 cells. The cells were treated with different doses (0, 2.5, 5, 10, 15, and 20 µM) of SGPF for 24 h. After incubation, cell viability of MH-S and HepG2 cells was detected by CCK-8 assay. Each experiment was repeated at least three times. (**a**) MH-S cells group, (**b**) HepG2 cells group, CCK-8: Cell Counting Kit-8.

MH-S and HepG2 cells were first treated with different concentrations (0–20 µM) of SGPF for 24 h, and then the effect of SGPF on cell viability was examined using the CCK-8 assay. As shown in Figure 2, the cell viability of MH-S and HepG2 cells treated with various concentrations of SGPF had no significant difference compared with the control group. The results indicated that SGPF had no effect on the viability of two cells; the percentages of viable cells were from 88.23% to 95.4% in the MH-S cell group and from 84.35% to 96.17% in the HepG2 cell group (Figure 2).

### 3.2. SGPF Inhibited LPS-Induced Inflammatory Cytokine Levels

**Figure 3:**
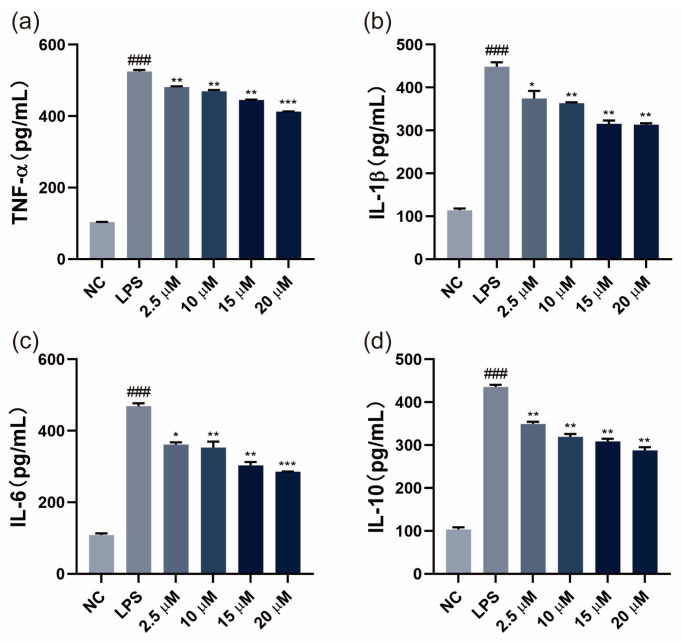
Effect of SGPF on release of LPS-treated inflammatory cytokines in MH-S cells. (**a**–**d**) The release of TNF-α, IL-1β, IL6, and IL-10 was detected by ELISA assay in MH-S cells. Different symbols above the bars indicate that the means of different groups were significantly different (*p* < 0.05) according to ANOVA. Each experiment was repeated at least twice. ^###^ *p* < 0.001 vs. control group; * *p* < 0.05, ** *p* < 0.01, *** *p* < 0.001 vs. LPS group.

SGPF exerted effects on the LPS-induced inflammatory cytokines TNF-α, IL-1β, IL-6, and IL-10 in MH-S cells with ELISA kits. The results revealed that the levels of TNF-α, IL-1β, IL-6, and IL-10 expression were markedly elevated in LPS-treated MH-S cells compared with the control group (*p* < 0.05). However, SGPF significantly reduced the levels of inflammatory cytokines in a dose-dependent manner (*p* < 0.05). These data indicated that SGPF could inhibit the overexpression of inflammatory cytokines (Figure 3).

### 3.3. Lipid Droplet Accumulation in OA-Induced HepG2 Cells

**Figure 4:**
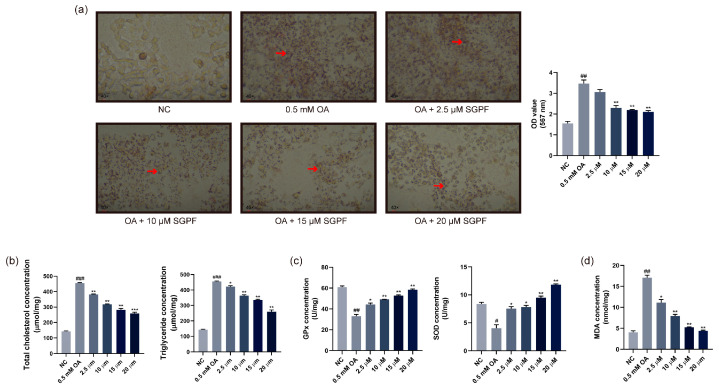
Effect of SGPF on OA-induced HepG2 cells. (**a**) The effect of SGPF on lipid accumulation induced by OA in HepG2 cells. (**b**) The effect of SGPF on intracellular total cholesterol and triglyceride content. (**c**,**d**) The effect of SGPF on intracellular antioxidant enzymes and MDA content. Different symbols above the bars indicate that the means of different groups were significantly different (*p* < 0.05) according to ANOVA. Each experiment was repeated at least twice. The red arrows showed lipid droplet aggregation. ^#^ *p* < 0.05, ^##^ *p* < 0.01, ^###^ *p* < 0.001 vs. control group; * *p* < 0.05, ** *p* < 0.01, *** *p* < 0.001 vs. LPS group.

After being pre-treated with different concentrations of SGPF for 24 h in HepG2 cells, 0.5 mM OA was administered to prompt lipid formation in the cells for 24 h. As shown in Figure 4a, the NC group produced almost no oil droplets, whereas the OA-induced group had a large aggregation of oil droplets. The number of red lipid droplets gathered in HepG2 cells treated with 0.5 mM OA for 24 h was significantly higher (^##^ *p* < 0.01) compared to the negative control group. However, to a certain extent, the intracellular lipids were reduced in a dose-dependent manner after treatment with SGPF. As shown in Figure 4a, these results displayed that the lipid-decreasing effect of SGPF became increasingly prominent with increasing concentration. The optimal lipid-lowering effect was achieved with 20 μM (** *p* < 0.01), which reduced 3/5 of the lipids in the model group.

### 3.4. Effect of SGPF on TC, TG, and Antioxidant Agent Levels in Cells

After treatment of HepG2 cells with 0.5 mM OA for 24 h, the intracellular TC level was significantly elevated (^###^ *p* < 0.001), being 3–4 times higher than that of the control group without treatment, whereas the group treated with SGPF exhibited a significant reduction in TC levels. As the concentration increased, the inhibition became increasingly evident, which 20 μM of SGPF achieving the better result (*** *p* < 0.001). Similarly, after treatment with SGPF, a noteworthy difference was observed between the administered group and the OA group (^###^ *p* < 0.001), and the former data showed a significant, dose-dependent reduction in intracellular TG content (Figure 4b).

As shown in Figure 4c, the OA-exposed group had much lower levels of GPx and SOD in HepG2 cells compared to the negative control group. In contrast, after SGPF treatment, the levels of intracellular GPx and SOD gradually rose with increasing drug concentration, and the best effect was observed when the concentration of SGPF was 20 μM (** *p* < 0.001). As shown in Figure 4d, the OA group, in comparison with the negative control, produced a large amount of MDA (^##^ *p* < 0.01). Meanwhile, a dose-dependent effect was observed, with 20 µM of SGPF lowering about 75% of MDA levels compared to the OA group, which has high antioxidant ability.

### 3.5. SGPF Inhibited OA-Induced Oxidative Stress in HepG2 Cells

**Figure 5:**
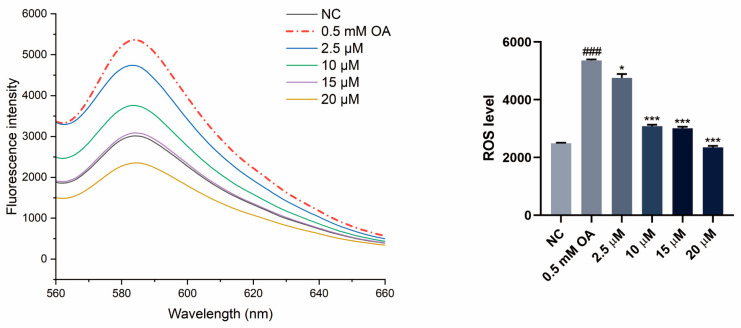
SGPF inhibited OA-induced oxidative stress in HepG2 cells. Different symbols above the bars indicate that the means of different groups were significantly different (*p* < 0.05) according to ANOVA. Each experiment was repeated at least twice. ^###^ *p* < 0.001 vs. control group; * *p* < 0.05, *** *p* < 0.001 vs. LPS group.

The effect of SGPF on intracellular ROS levels was also examined using a fluorescence spectrophotometer, and then the effect of SGPF on HepG2 cell oxidative stress was assessed. Compared with the negative control group, as shown in Figure 5, OA administration significantly increased ROS production (^###^ *p* < 0.001). Next, it was observed that different concentrations of SGPF reduced oxidative ROS production in HepG2 cells when compared with the OA group (* *p* < 0.05). Among these concentrations, it was observed that 20 µM had the highest efficacy, whereas 10 µM and 15 µM had nearly identical effects, all of which were statistically significant (*** *p* < 0.001).

### 3.6. SGPF Treatment Suppressed TLR4/NF-κB/MyD88 Pathway Activation in LPS-Induced MH-S Cells

**Figure 6:**
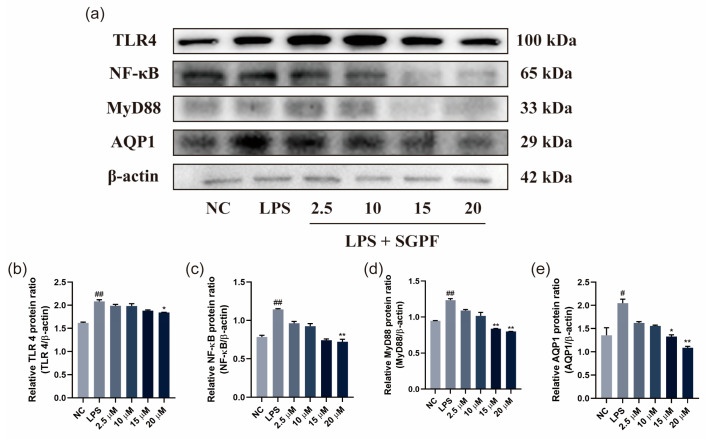
Effect of SGPF on protein expression levels of TLR4/ NF-κB/MyD88 pathway. Signaling molecule expression, including TLR4, NF-κB, MyD88, and AQP1 (**a**). The expression level of TLR4 (**b**), NF-κB (**c**), MyD88 (**d**), and AQP1 (**e**) in LPS-induced MH-S cells. Different symbols above the bars indicate that the means of different groups were significantly different (*p* < 0.05) according to ANOVA. Each experiment was repeated at least twice. ^#^ *p* < 0.05, ^##^ *p* < 0.01 vs. control group; * *p* < 0.05, ** *p* < 0.01 vs. LPS group. Original images of (**a**) can be found in Figure S6.

The protein levels of TLR4, NF-κB, and MyD88 in each group were determined by Western blotting, and the results are shown in Figure 6. Compared with the NC group, the protein levels of inflammatory expression in the 100 ng/mL LPS group were increased to varying degrees; correspondingly, the expression of TLR4, NF-κB, MyD88, and AQP1 was significantly increased (^##^ *p* < 0.01). However, the SGPF intervention reversed this change. In contrast, the TLR4, NF-κB, MyD88, and AQP1 protein concentrations in the SGPF group were significantly decreased compared to the LPS group (** *p* < 0.01). 

### 3.7. SGPF Treatment Increased Nrf2/HO-1 Pathway Activation in OA-Induced HepG2 Cells

**Figure 7:**
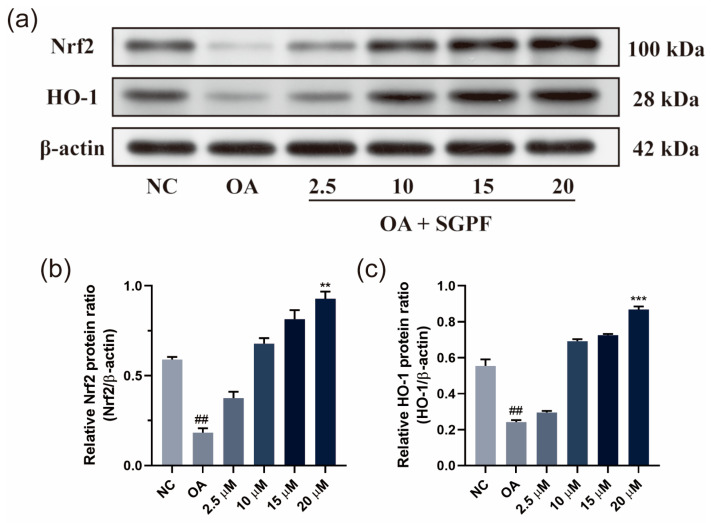
Effect of SGPF on protein expression levels of Nrf2/HO-1 pathway. Signaling molecule expression (**a**). The expression level of Nrf2 (**b**) and HO-1 (**c**) in OA-induced HepG2 cells. Different symbols above the bars indicate that the means of different groups were significantly different (*p* < 0.05) according to ANOVA. Each experiment was repeated at least twice. ^##^ *p* < 0.01 vs. control group; ** *p* < 0.01, *** *p* < 0.001 vs. LPS group. Original images of (**a**) can be found in Figure S7.

To investigate the potential mechanism, the protein expressions of Nrf2 and HO-1 were detected. As shown in Figure 7, the protein expression of Nrf2/HO-1 in 0.5 mM OA-stimulated HepG2 cells was remarkably decreased compared with that in negative control cells (^##^ *p* < 0.01), and the administration of SGPF (in different concentrations) further promoted the expression of Nrf2 compared with the 0.5 mM OA group (** *p* < 0.01). Moreover, the expression of HO-1 was found to be upregulated in the SGPF group compared with the 0.5 mM OA group (*** *p* < 0.001). The incubation of SGPF at a lower concentration also increased the expression of Nrf2 and HO-1.

## 4. Discussion

The aim of this study was to elucidate the protective effect of SGPF against LPS-stimulated cellular inflammation and its underlying mechanisms, thereby providing a promising candidate therapy for asthma patients. Interestingly, the flavonoid glycoside not only showed remarkable anti-inflammatory activity against MH-S cells but also inhibited liver injury in NAFLD. This indicates that SGPF could potentially serve as an anti-inflammatory and hepatoprotective compound in *Siraitia grosvenorii*. This study showed for the first time the anti-inflammatory potential of SGPF utilizing LPS-induced MH-S cells. Macrophages, a crucial component of the immune system, can release a diverse range of inflammatory cytokines, including TNF-α, IL-1β, and IL-6. Further examination of their expression revealed that SGPF was able to inhibit the expression of TNF-α, IL-1β, IL-6, and IL-10 in a dose-dependent manner, which was in agreement with previous studies [25,26]. TNF-α has been described as a circulating factor that can lead to tumor necrosis and has been identified as a key regulator of inflammatory action [27]. During the inflammatory process, the presence of IL-1β and IL-6 might intensify the inflammatory response by facilitating the recruitment and activation of neutrophils [28]. The inflammatory cytokines IL-1β, IL-6, and IL-10 have an important role in the inflammatory process, in which expression levels and action mechanisms contribute to the comprehension of disease pathogenesis and therapeutic approaches. The TLR4/NF-κB/MyD88 pathway has been recognized as a central link in the pathogenic process of systemic inflammation [29]. In the present study, on the one hand, TLR4/MyD88 activation has been found to be notably upregulated in LPS-induced MH-S cells, leading to increased expression of pro-inflammatory cytokines and related chemokines, including TNF-a, IL-1β, IL-6, and IL-10. Uncontrolled expression of inflammatory cytokines is an important process in the pathogenesis of asthma [30]. On the other hand, LPSs could raise the levels of inflammatory cytokines in cells, indicating that TLR4/MyD88-activated signaling is involved in LPS-induced inflammatory injury. After SGPF treatment, cells produced fewer inflammatory cytokines. In the TLR4/NF-κB/MyD88 pathway, SGPF significantly inhibited TLR4 protein expression and the activation of the TLR4/MyD88 pathway in a dose-dependent manner. The above results suggest that SGPF exerts an inhibitory effect on LPS-induced inflammation in MH-S cells via suppressing TLR4/NF-κB/MyD88 signaling pathway activation. The relationship between asthma and the TLR4/NF-κB/MyD88 signaling pathway is complex and diverse, involving the regulation of multiple immune responses and inflammatory responses. Pharmaceutical development targeting different segments of this pathway is expected to provide new ideas for asthma treatment.

Oxidative stress is an imbalance between the oxidative and antioxidant capacities within the body and excessive ROS accumulation. ROS overaccumulation is indeed an important factor in NAFLD pathogenesis, leading to mitochondrial dysfunction, which further triggers hepatocellular injury and activates the inflammatory response [31,32]. There is growing evidence that in NAFLD, ROS activates inflammatory action by stimulating the release of inflammatory cytokines that further trigger hepatocyte injury and fibrosis [33,34]. Nrf2 belongs to the CNC-bZIP family and serves as an essential part in the antioxidant mechanism, which triggers the expression of antioxidant genes to counteract oxidative damage. Previous studies have shown that Nrf2 is a potential target for the alleviation of NAFLD [35]. Under controlled conditions, Nrf2 binds to related proteins and exists in an inactive state in the cytoplasm. However, when activated by a stimulus, Nrf2 detaches and translocates to the nucleus, binds to the ARE, and then activates the transcription of downstream antioxidant genes, which could assist in reducing ROS levels and inhibiting oxidative stress [36,37]. HO-1 attenuates oxidative damage and serves as a downstream gene for Nrf2 [38]. In this study, we systematically investigated the anti-NAFLD effect of SGPF and the underlying mechanism in OA-induced HepG2 cells. In healthy cell groups, the oxidative and antioxidant systems are in a dynamic equilibrium and intracellular antioxidant mechanisms can effectively scavenge ROS. However, under OA-induced conditions, excessive TC, TG, and lipid accumulation in HepG2 cells introduces lipotoxicity, leading to mitochondrial dysfunction and ROS production [39]. Moreover, lipid overload also leads to oxidative stress damage and intracellular MDA production, which exceeds the antioxidant ability of the organism and inhibits SOD production. Our results suggested that SGPF lowers ROS overproduction by OA-induced and oxidative damage via triggering the Nrf2/HO-1 signaling pathway. Furthermore, SGPF suppressed OA-induced inflammation as it reduced the overproduction of oxidation-related agents through the Nrf2 signaling chain. SGPF also reversed SOD activity and decreased GSH content as well as excess ROS and MDA.

## 5. Conclusions

**Figure 8:**
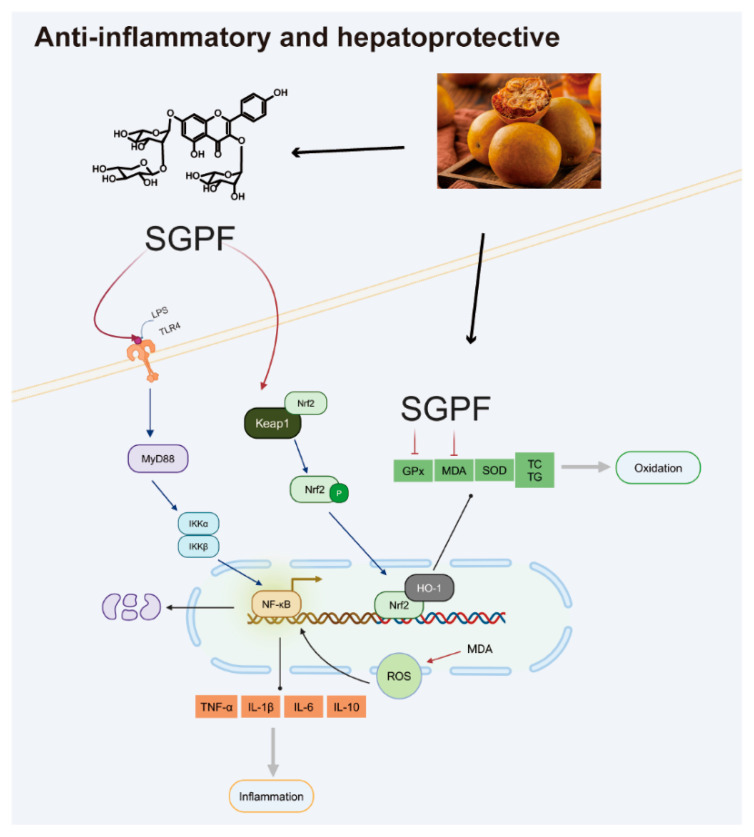
The SGPF for anti-inflammatory and hepatoprotective effects.

To sum up, the findings of our study demonstrate that the flavonoid glycoside SGPF, isolated for the first time from *S. grosvenorii*, was involved in the regulation of macrophage anti-inflammation via targeting the TLR4/NF-κB/MyD88 signaling cascade by causing LPS-induced inflammatory macrophages to reduce the release of the inflammatory cytokines TNF-α, IL-1β, IL-6, and IL-10. In addition, the results of another study show that oxidative stress leads to inflammasome activation. Being a flavonoid, SGPF shows higher antioxidant potential, which highlighted its hepatoprotective activity. The antioxidant protection of SGPF was efficient in suppressing intracellular antioxidant enzyme (GPx and MDA) content, preventing SOD depletion, and reducing intracellular ROS, which was very remarkable in HepG2 cells. After SGPF administration, Nrf2 and HO-1 were highly expressed in the OA-induced HepG2 cell model, which might be a key process in OA pathogenesis. Consequently, there is reason to speculate that targeting Nrf2/HO-1 signaling could be an appealing and prospective therapeutic strategy to prevent OA-induced inflammation. This is the first study on SGPF that exhibited its anti-inflammatory and hepatoprotective potential. Finally, a flavonoid that is abundant in a number of sugar groups in *S. grosvenorii*, and mogroside V, which is used as a sweetener in this fruit, might also have some connection; more research is needed to further promote therapeutic feeding. *S. grosvenorii*, a traditional and cost-effective food and medicinal plant, warrants further exploration for these compounds’ anti-inflammatory and hepatoprotective potential for enhanced contributions to human health (Figure 8).

## Data Availability

The data presented in this study are available on request from the corresponding authors (data are part of an ongoing study).

## Supplementary Materials

The following supporting information can be downloaded at: https://www.mdpi.com/article/10.3390/biom14040450/s1, Figure S1: ^1^H-NMR of SGPF; Figure S2: ^13^C-NMR of SGPF; Figure S3: ^1^H-^1^H COSY of SGPF; Figure S4: HSQC of SGPF; Figure S5: HMBC of SGPF; Figure S6: Original images of Figure 6A; Figure S7: Original images of Figure 7A.

## Abbreviations

DCM	Dichloromethane	MDA	Malondialdehyde	RPMI	Roswell Park Memorial Institute
EA	Ethyl acetate	NAFLD	Non-alcoholic fatty liver disease	SGPF	*Siraitia. grosvenorii* pomace flavonoid
FBS	Fetal bovine serum	n-BuOH	n-Butanol	SOD	Superoxide dismutase
HO-1	Heme oxygenase-1	NC	Negative control	TLC	Thin-layer chromatography
IL-1β	Interleukin-1β	NHGR	*S. grosvenorii* residual extract	TNF-α	Tumor necrosis factor-α
IL-6	Interleukin-6	Nrf2	Nuclear factor erythroid-2-related factor-2	VEGF	Vascular endothelial growth factor
LPSs	Lipopolysaccharides	OA	Oil acid		
MeOH	Methanol	PE	Petroleum benzin		
