# Supplementary material for: A Flavonoid Glycoside Compound from Siraitia grosvenorii with Anti-Inflammatory and Hepatoprotective Effects In Vitro
Source: Biomolecules. 2024 Apr 7;14(4):450. doi: 10.3390/biom14040450 (PMC11048398; doi:10.3390/biom14040450)
Supplement: Supplementary file 1 [file biomolecules-14-00450-s001.zip › biomolecules-2921818-Figure S5,S6.pdf]

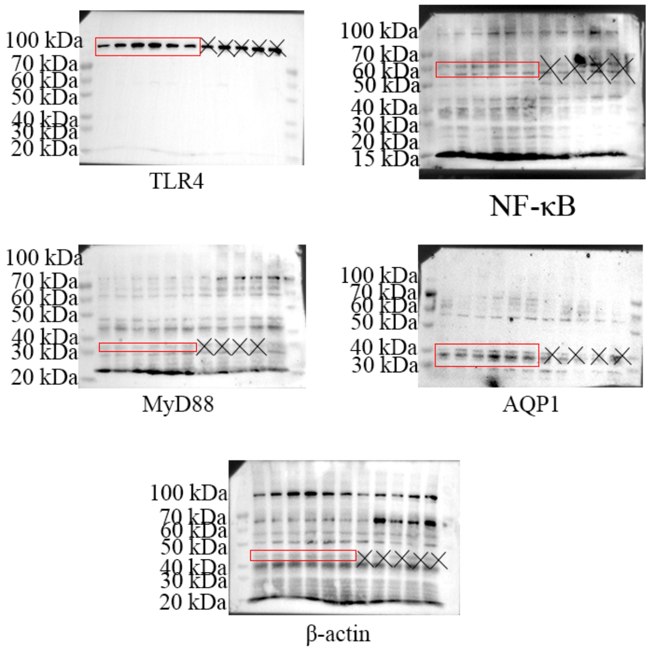

Figure S6 The samples derive from the same experiment and that blots were processed in parallel.

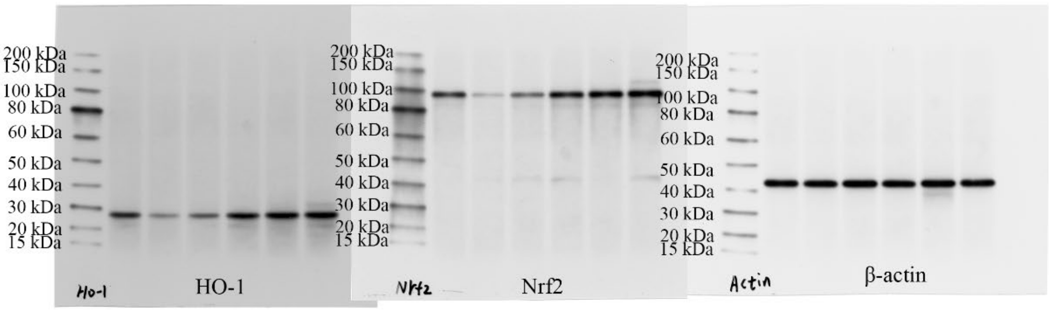

Figure S7 The samples derive from the same experiment and that blots were processed in parallel.
