# Supplementary material for: A Flavonoid Glycoside Compound from Siraitia grosvenorii with Anti-Inflammatory and Hepatoprotective Effects In Vitro
Source: Biomolecules. 2024 Apr 7;14(4):450. doi: 10.3390/biom14040450 (PMC11048398; doi:10.3390/biom14040450)
Supplement: Supplementary file 1 [file biomolecules-14-00450-s001.zip › biomolecules-2921818-supplementary.pdf]

## Supplementary Materials

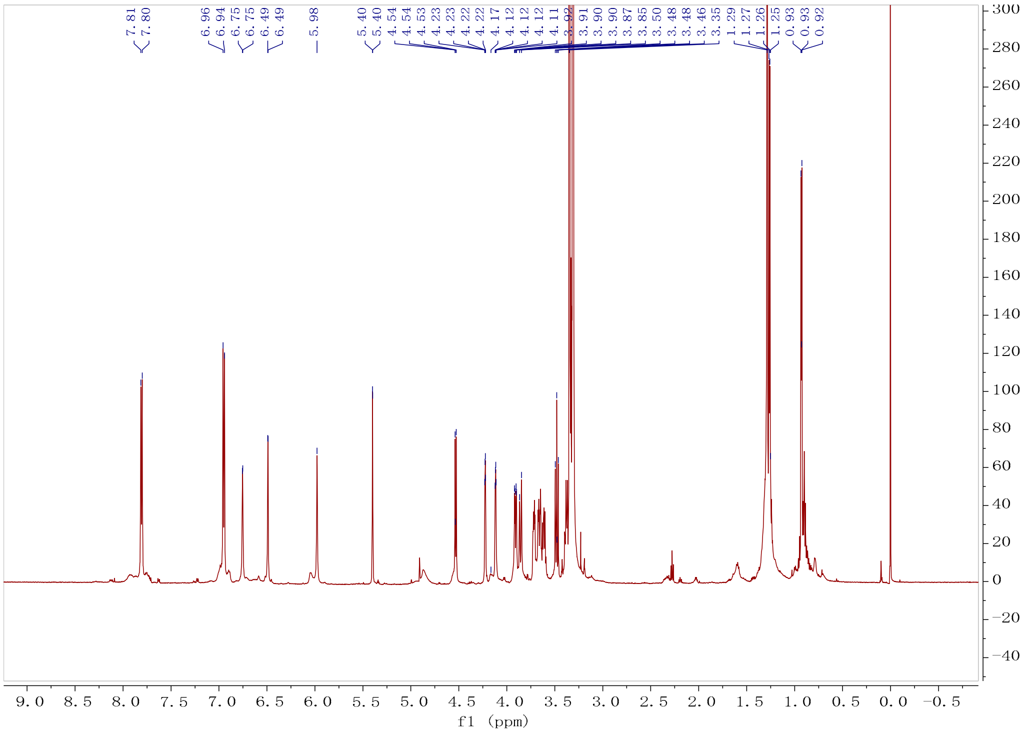


**Figure S1.** $^{1}$H-NMR of SGPF

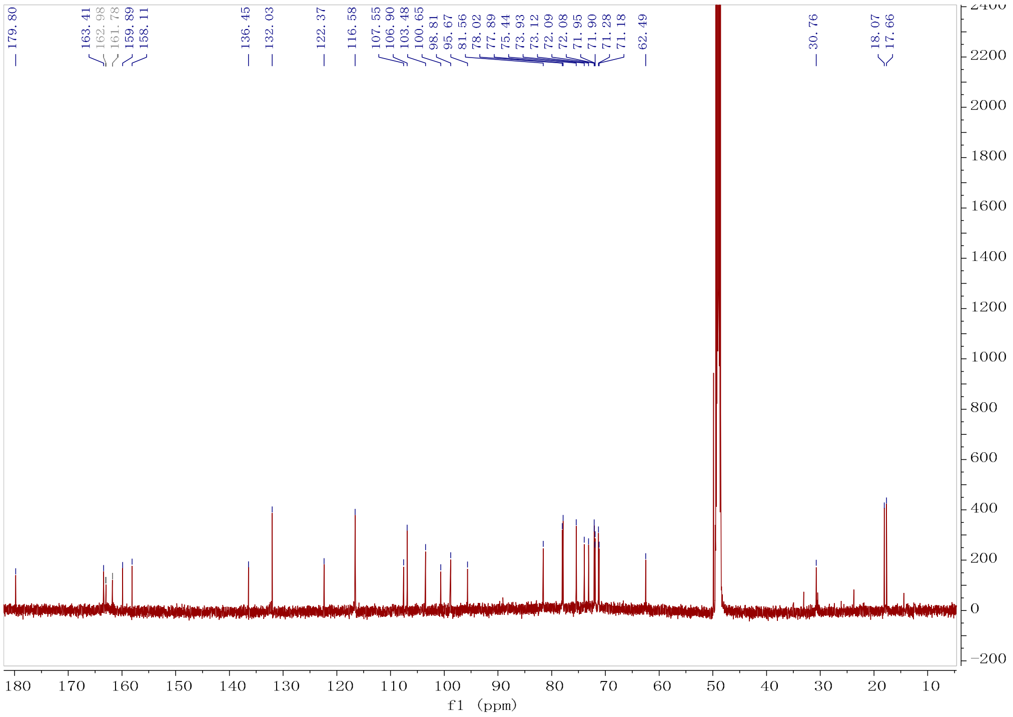


**Figure S2.** 13C-NMR of SGPF

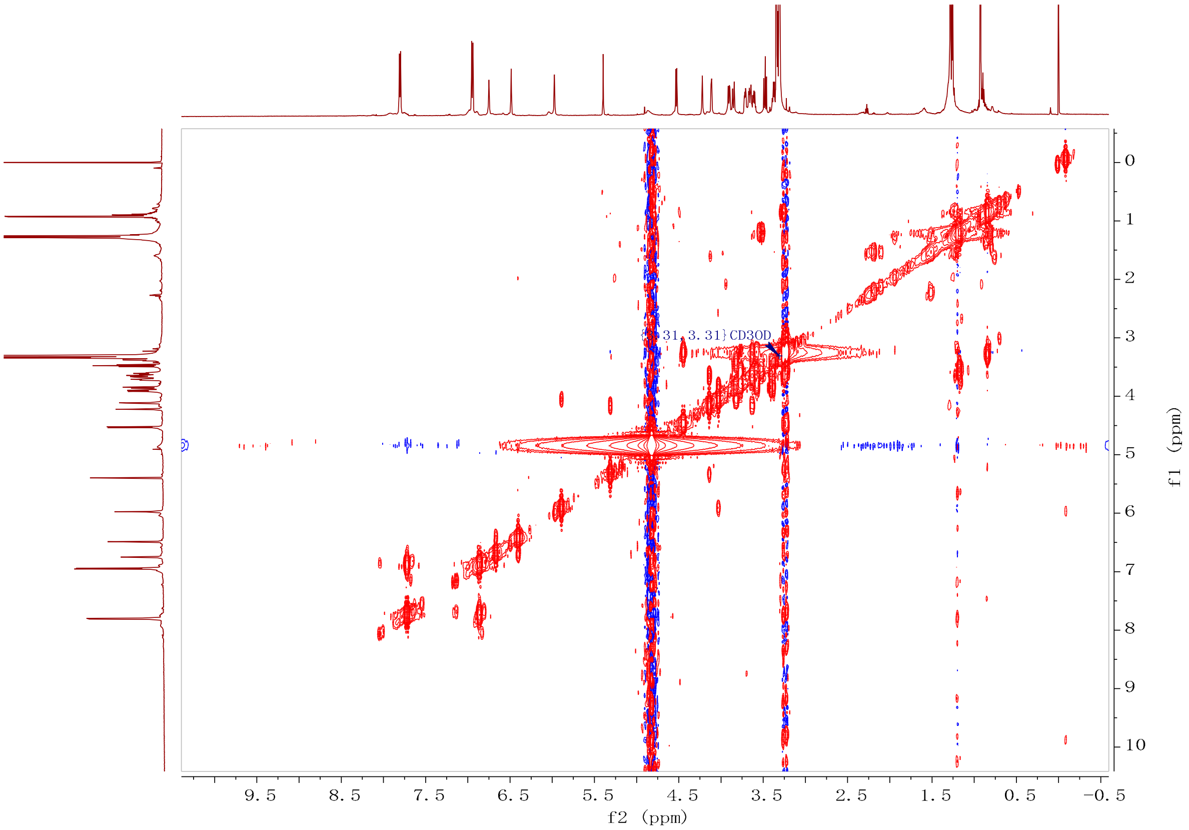


**Figure S3.** 1H-1H COSY of SGPF

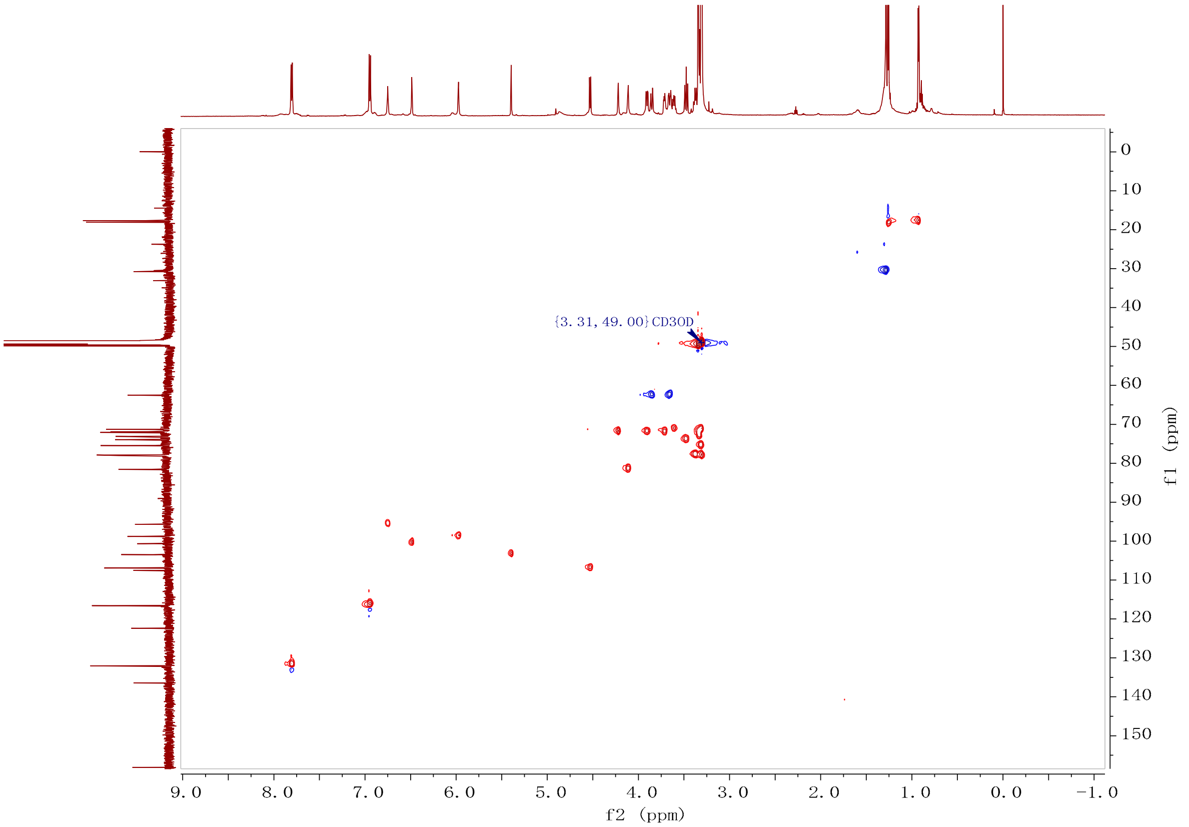


**Figure S4.** HSQC of SGPF

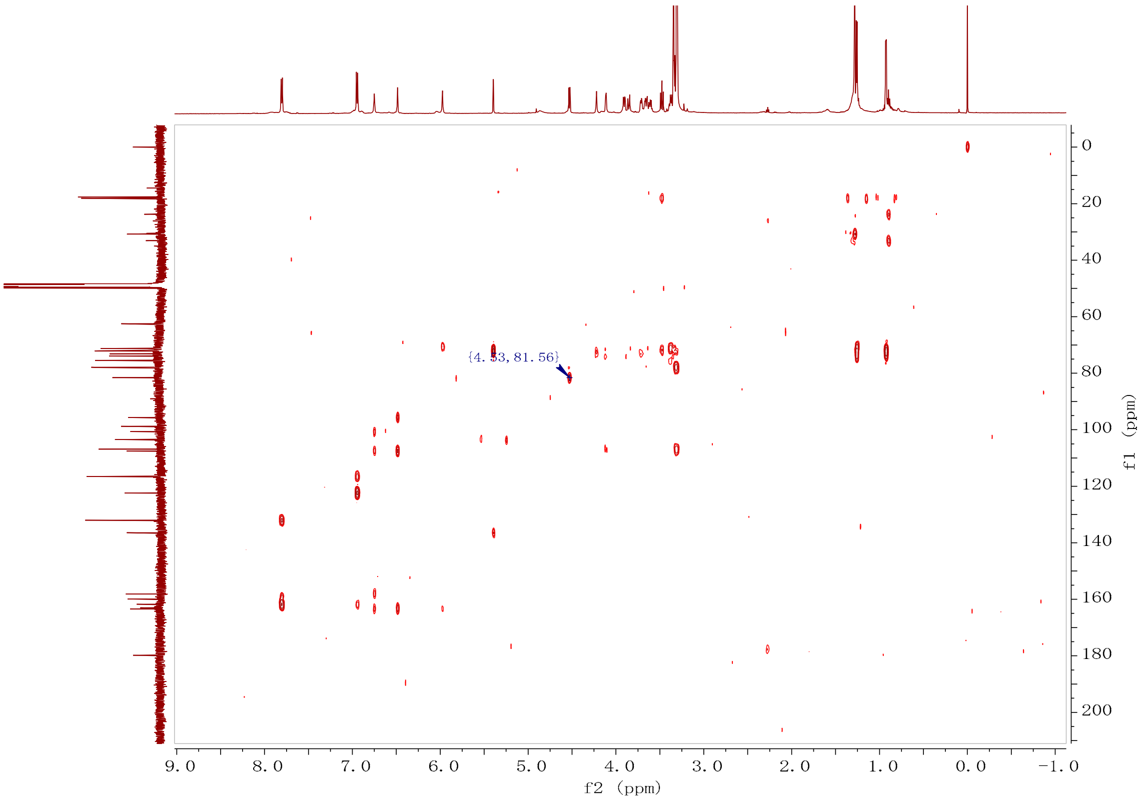


**Figure S5.** HMBC of SGPF
